# First Resistance Mechanisms Characterization in Glyphosate-Resistant *Leptochloa virgata*

**DOI:** 10.3389/fpls.2016.01742

**Published:** 2016-11-18

**Authors:** Ricardo Alcántara-de la Cruz, Antonia M. Rojano-Delgado, María J. Giménez, Hugo E. Cruz-Hipolito, José A. Domínguez-Valenzuela, Francisco Barro, Rafael De Prado

**Affiliations:** ^1^Department of Agricultural Chemistry and Edaphology, Campus of Rabanales, University of CordobaCordoba, Spain; ^2^Institute for Sustainable Agriculture, Spanish National Research CouncilCordoba, Spain; ^3^Bayer CropScience MexicoMexico City, Mexico; ^4^Department of Agricultural Parasitology, Chapingo Autonomous UniversityTexcoco, México

**Keywords:** 5-enolpyruvyl shikimate-3-phosphate synthase, EPSPS gene, Pro-106 substitution, reduced absorption and translocation, tropical sprangletop.

## Abstract

*Leptochloa virgata* (L.) P. Beauv. is an annual weed common in citrus groves in the states of Puebla and Veracruz, Mexico limiting their production. Since 2010, several *L. virgata* populations were identified as being resistant to glyphosate, but studies of their resistance mechanisms developed by this species have been conducted. In this work, three glyphosate-resistant populations (R8, R14, and R15) collected in citrus orchards from Mexico, were used to study their resistance mechanisms comparing them to one susceptible population (S). Dose-response and shikimic acid accumulation assays confirmed the glyphosate resistance of the three resistant populations. Higher doses of up to 720 g ae ha^-1^ (field dose) were needed to control by 50% plants of resistant populations. The S population absorbed between 7 and 13% more ^14^C-glyphosate than resistant ones, and translocated up to 32.2% of ^14^C-glyphosate to the roots at 96 h after treatment (HAT). The R8, R14, and R15 populations translocated only 24.5, 26.5, and 21.9%, respectively. The enzyme activity of 5-enolpyruvyl shikimate-3-phosphate synthase (EPSPS) was not different in the S, R8 and R14 populations. The R15 Population exhibited 165.9 times greater EPSPS activity. Additionally, this population showed a higher EPSPS basal activity and a substitution in the codon 106 from Proline to Serine in the EPSPS protein sequence. EPSPS gene expression in the R15 population was similar to that of S population. In conclusion, the three resistant *L. virgata* populations show reduced absorption and translocation of ^14^C-glyphosate. Moreover, a mutation and an enhanced EPSPS basal activity at target-site level confers higher resistance to glyphosate. These results describe for the first time the glyphosate resistance mechanisms developed by resistant *L. virgata* populations of citrus orchards from Mexico.

## Introduction

Glyphosate is the most extensively used systemic foliar herbicide of broad spectrum used for over 40 years (early 1970s) to control of annual and perennial weeds (Gomes et al., 2014; Armendáriz et al., 2016). The target-site of glyphosate is the 5-enolpyruvyl shikimate-3-phosphate synthase (EPSPS; EC 2.5.1.19), an intermediate in the shikimate pathway (Gomes et al., 2014). EPSPS inhibition by glyphosate does not allow the synthesis of phenylalanine, tyrosine, and tryptophan resulting in accumulation of shikimic acid and depletion of aromatic amino acid pools (Gomes et al., 2014; Maroli et al., 2016). Therefore, glyphosate acts rapidly in reducing photosynthesis activity, carbon metabolism, mineral nutrition, and oxidative events, and to disturb plant–microorganism interactions (Gomes et al., 2014). To date 36 weed species have been reported as being resistant to glyphosate with more than 266 cases (Heap, 2016).

Resistance to glyphosate could be caused by different mechanisms either in the target or non-target site (Salas et al., 2015). Both groups limiting the amount of glyphosate which reaches the EPSPS at toxic levels, or causing a loss of affinity between the EPSPS and the glyphosate (Powles, 2010). Target-site resistance (TSR) mechanisms includes amino acid substitutions in DNA sequence, represented by nucleotide changes in Thr-102 and Pro-106 positions, that synthesizes the EPSPS enzyme (Sammons and Gaines, 2014; Chen et al., 2015; Yu et al., 2015; Alcántara-de la Cruz et al., 2016b); multiple EPSPS gene copy numbers resulting in a higher amplification, expression and activity of the EPSPS (Gaines et al., 2013; Vila-Aiub et al., 2014). To date, glyphosate is the only herbicide known to evolve amplification of the target-site (EPSPS) conferring resistance with field applications (Ribeiro et al., 2014).

Non-target-site resistance (NTSR) mechanisms can confer unpredictable resistance to herbicides (Déyle, 2013). Some are: limited glyphosate leaf absorption (Vila-Aiub et al., 2012); reduced glyphosate translocation to meristematic zones, mainly restricted within the treated leaves (Shaner, 2009; Yanniccari et al., 2012); sequestration of glyphosate in the vacuole (Ge et al., 2012); and glyphosate degradation to non-toxic compounds (sarcosine, glyoxylate, formaldehyde and amino methyl phosphonate) (Duke, 2011) resulting in significantly less glyphosate throughout the whole plants.

Weeds can evolve several herbicides resistance mechanisms, and the individuals of the same species can express different mechanism(s) (Alarcón-Reverte et al., 2015). In *Leptochloa virgata* (L.) P. Beauv., studies about of glyphosate efficacy by dose-response assay, genetic diversity characterization using inter simple sequence repeat (ISSR) markers of different glyphosate-resistant *L. virgata* populations (Alcántara-de la Cruz et al., 2016c), and field trials to provide alternative herbicides for their control have been carried out (Pérez-López et al., 2014). However, no studies on the glyphosate resistance mechanisms involved in their resistance have been conducted, and currently there are no reports of others resistant populations of this species elsewhere in the world.

Based on previous reports, and the lack of any knowledge on the glyphosate resistance mechanisms of *L. virgata*, we hypothesized that the resistance mechanisms developed by *L. virgata* plants could be similar to the mechanisms evolved by others glyphosate resistant species. Therefore, the objective of this work was characterize for the first time whether the glyphosate resistance developed by three resistant *L. virgata* populations was due either target or non-target site mechanisms, comparing them to one susceptible population (S). These populations were used as being representatives of the species in citrus cropping systems from Mexico.

## Materials and Methods

### Plant Material and Growing Conditions

Seeds of four *L. virgata* populations (S, R8, R14, and R15) collected in Persian lime groves from Veracruz, previously characterized with different levels of susceptibility to glyphosate by Alcántara-de la Cruz et al. (2016c), were used. Individuals of the *L. virgata* populations studied had a similar glyphosate response glyphosate within them. Glyphosate resistant R14 and R15 populations from Cuitláhuac municipality, were selected for being geographically close (18.75° N, 96.53° W), but genetically different. Also, the most glyphosate resistant population (R8) from Martínez de la Torre municipality (20.16° N, 97.07° W) was included in this study. The S population was used as control (18.79° N, 96.69° W).

The seeds were sown on trays with peat saturated at field capacity. The trays were covered with a layer of plastic until emergence and placed in a growth chamber at 26/18°C (day/night) with a photoperiod of 16 h at 850 mmol m^-2^ s^-1^ of light density and 60% relative humidity. Germinated seedlings were individually transplanted into plastic pots in 250 mL of substrate (sand and peat 1:1). Subsequently, the pots were placed in a growth chamber under the conditions described above and watered daily.

### Dose-Response Assays

Plants of the four populations with 3–4 true leaves were treated with the following glyphosate rates: 0, 45, 90, 180, 360, 720, 1040, 1440, and 1800 g ae ha^-1^. Glyphosate (Roundup Energy 45% w/v, Monsanto, Spain) applications were carried out in a treatment chamber (Devries Manufacturing, Hollandale, MN, USA) equipped with TeeJet 8002EVS flat fan nozzle calibrated at 200 kPa, a height of 50 cm and 200 L ha^-1^ of application volume. The plants were harvested at ground level 21 days after treatment (DAT) and stored separately in paper envelopes. The samples were dried in an oven (JP Selecta S.A., Barcelona, Spain) at 60°C for 4 days and weighed to determine the dry weight. Data were expressed as percentage of dry weight compared to the untreated control plants. The experiment was repeated twice in a completely randomized design with 10 replicates per dose.

### Shikimic Acid Accumulation

Samples of 50 mg (leaf disks 4 mm in diameter) of plant tissue were taken from young leaves of three plants with 3–4 true leaves of each *L. virgata* population according to Dayan et al. (2015). The disks were placed in 2 mL-Eppendorf tubes containing 999 μL of monoammonium phosphate (NH_4_H_2_PO_4_ 10 mM, pH 4.4). Volumes of 1 μL of glyphosate solutions at different concentrations were added (0, 0.1, 1, 10, 50, 100, 200, 400, 600, and 1000 μM). The samples were incubated for 24 h in the growth chamber under growing conditions described above. Next, they were incubated at 60°C for 30 min. Volumes of 250 μL of HCl 1.25 N were added and incubated at 60°C for 15 min. Aliquots of 250 μL were transferred to new tubes adding 500 μL of periodic acid (0.25% w/v) and sodium metaperiodate (0.25 % w/v) in a 1:1 ratio. The samples were incubated at room temperature (25°C) during 90 min, and next, 500 μL of a mix of sodium hydroxide (NaOH 0.6 N) plus sodium sulfite (Na_2_SO_3_ 0.22 N) in a 1:1 ratio was added and mixed.

The experiment was arranged in a completely randomized design with three technical replications by sample of each population for each glyphosate concentration, and the study was repeated twice. A standard curve was done using known concentrations of shikimate. The absorbance of samples was measured at 382 nm in a spectrophotometer (DU-640, Beckman Coulter Inc., Fullerton, CA, USA). The absorbance values were converted into mg of shikimic acid per g of fresh weight.

### Absorption and Translocation

Plants with 3–4 true leaves from the four *L. virgata* populations were treated with a solution of ^14^C-glyphosate [glycine-2-^14^C] (specific activity 273.8 MBq mmol^-1^, American Radiolabeled Chemicals, Inc., Saint Louis, MO, USA) + commercial glyphosate. The solution applied contained a specific activity of 0.834 kBq μL^-1^ and a glyphosate concentration of 1.8 g ea L^-1^ (360 g ea ha^-1^ in 200 L). One drop of 1 μL plant^-1^ of solution was applied with a micropipette (Lab Mate HTL, Matosinhos, Portugal) on the adaxial surface of the first-second leaf. After treatment, the plants were maintained in the growth chamber at the growing conditions described above. At 24, 48, 72, and 96 HAT, the treated leaves were washed three times separately with 1 mL of water-acetone (1:1 v/v) to recover the non-absorbed ^14^C-glyphosate. The washing solution was mixed with 2 mL of scintillation liquid (Ultima Gold, Perkin-Elmer, BV BioScience Packard), and analyzed by liquid scintillation spectrometry (LSS) in a scintillation counter (LS 6500, Beckman Coulter Inc., Fullerton, CA, USA) during 10 min per sample. The whole plants were carefully removed from the pot and washed, mainly the roots. The plants were individually divided into treated leaf, remainder of the plant and root. The samples were stored in flexible combustion cones (Perkin-Elmer, BV BioScience Packard), dried in an oven at 60°C for 4 days. Next, the samples were combusted in a biological oxidizer (Packard Tri Carb 307, Packard Instrument Co., Downers Grove, IL, USA). The CO_2_ released from the combustion was captured in 18 mL of a mix of Carbo-Sorb E and Permafluor (1:1 v/v) (Perkin-Elmer, BV BioScience Packard). The radioactivity of each individual sample was quantified by LSS during 10 min per sample. The experiment was arranged in a completely random design with five replicates per population at each time evaluated. The radioactive values were used to calculate recovery percentage as: [(kBq in treated leaf + kBq in plant + kBq in roots + kBq from washes)/kBq total applied] × 100. The average total recovery of ^14^C-glyphosate applied was >96% to the S, R8, and R14 populations, and <93% from the R15 population.

To visualize the ^14^C-glyphosate translocation, three whole plants per population at each visualization time (24, 48, 72, and 96 HAT) were treated under the same conditions as in the previous assay. The plants were washed individually, fixed on filter paper (25 cm × 12.5 cm) and dried at room temperature for 1 week. The samples were placed for 4 h beside a phosphor storage film (Storage Phosphor System: Cyclone, Perkin–Elmer Packard BioScience BV). A phosphor imager (Cyclon, Perkin-Elmer, Packard BioScience BV) was used to reveal the translocation.

### Enzyme Activity of the EPSPS

Plants of the four *L. virgata* populations were grown in pots (25 cm in diameter × 15 cm high: four plants per pot) under greenhouse conditions, in temperatures ranging from 17 to 31°C, and a photoperiod of 16 h. The natural light was complemented by 900 μmol^-2^ s^-1^ photosynthetic photon flux density delivered by incandescent and fluorescent lights. Samples of 5 g of foliar tissue from each population were obtained from the second and third youngest totally expanded leaves.

The methodology described by Sammons et al. (2007) was used for EPSPS extraction. The total soluble protein (TPS) in the extract was measured using a Kit for Protein Determination (Sigma-Aldrich, Madrid, Spain) following the manufacturer’s instructions. The specific EPSPS activity in plants from *L. virgata* populations was studied in the presence and absence (basal activity) of glyphosate. The EPSPS activity was determined using a EnzChek Phosphate Assay Kit (Invitrogen, Carlsbad, CA, USA) following the manufacturer’s instructions. The glyphosate concentrations used were: 0, 1, 10, 100, 1000, 10000 μM. Three replicates at each glyphosate concentration were analyzed. The release of phosphate on the bottom level was measured during 10 min at 360 nm in a spectrophotometer (DU-640, Beckman Coulter Inc., Fullerton, CA, USA).

### EPSPS Gene Sequencing

Samples of young leaf tissue (≈100 mg) from 5 glyphosate-susceptible (S) and 15-resistant (R8, R14, and R15) *L. virgata* individuals were taken, and stored at -80°C for RNA extraction. Total mRNA was isolated following the methodology described by Pistón (2013). Integrity of RNA was verified in 0.8% agarose gel and quantified in a NanoDrop ND-1000 spectrophotometer (Thermo Scientific, Walthman, MA, USA). First strand complementary DNA (cDNA) synthesis was carried out using 1 μg of RNA in all the samples. An iScript cDNA Synthesis Kit (Bio-Rad Laboratories, Inc. Hercules, CA, USA) was employed following the manufacturer’s instructions. Lv-F3 and Lv-R2 primers (**Table 1**) were designed using the software Primers3Plus^1^, based on conserved regions of the EPSPS gene sequences from *Eleusine indica* (GenBank Accession: AY157642.1, HQ403647.1) and *Lolium multiflorum* (GenBank Accession: DQ153168.2). Individual PCR reactions were carried out using cDNA from each sample of the S and R15 populations. Each PCR reaction was performed using 50 ng of cDNA, 0.2 μM of each primer, 0.2 mM dNTP mix (PE Applied Biosystems; Life Technologies S.A., Madrid, Spain), 2 mM MgCl_2_, 1X buffer, and 0.625 units of a 100:1 enzyme mixture of non-proofreading (*Thermus thermophilus*) and proofreading (*Pyrococcus furiosus*) polymerases (BIOTOOLS, Madrid, Spain) in a final volume of 25 μL. The PCR conditions were: 1 cycle of 94°C for 5 min; followed by35 cycles at 94°C for 30 s, 55°C for 30 s, and 72°C for 1 min; and a final extension at 72°C for 10 min. PCR products (10 μL) were checked by 1% agarose gel to corroborate amplification. The PCR products were ligated using the pGEM-T Easy Vector System (Promega Biotech Ibérica, SL, Madrid, Spain) following the manufacturer’s instructions, and cloned into competent cells of *E. coli* DH5α. Positive transformants were selected and fragment insertion confirmed through PCR using the M13F and M13R primers (**Table 1**). A total volume of 15 μL per sample containing 0.2 μM of each primer, 0.2 mM dNTP mix (PE Applied Biosystems; Life Technologies S.A., Madrid, Spain), 2 mM MgCl_2_, 1X buffer, and 0.625 units of non-proofreading (*Thermus thermophilus*) polymerase (BIOTOOLS, Madrid, Spain). The PCR conditions were: 1 cycle of 94°C for 5 min; followed by 28 cycles of 94°C for 30 s, 50°C for 30 s, and 72°C for 1 min; and a final extension at 72°C for 7 min. The plasmids were purified with the ilustra plasmidPrep Mini Spin kit (GE Healthcare, Buckinghamshire, UK), following the manufacturer’s instructions. Sanger sequencing was carried out by the STABVIDA sequencing service (Caparica, Portugal). A total of 30 clones from each population were sequenced. The assembly of the sequences was carried out by SeqMan Pro (Version 11, DNASTAR; Madison, WI, USA) and Geneious (Version 8.1.8, Biomatters Ltd, Auckland, New Zealand) software’s.

**Table 1:** Names and sequences of primers used in EPSPS gene sequencing and expression analysis of *L. virgata* populations.

Name	Sequence (5′ to 3′)
**EPSPS gene sequencing and cloning**	
Lv-F3^a^	AAGAGCTGTWGTYGTTGGCTG
Lv-R2^a^	AATAGCACCTCGCACTTGAG
M13F	CGCCAGGGTTTTCCCAGTCACGAC
M13R	TCACACAGGAAACAGCTATGAC

**qPCR**	
qLv-For1^a^	GGCAGGTTCCCGATTGARAA
qLv-Rev1^a^	YGCATTTCCACCAGCAGCTA
ADP-RF(a) For	TCTCATGGTTGGTCTCGATG
ADP-RF(a) Rev	GGATGGTGGTGACGATCTCT
β-Act F1^a^	ATGGTAGGGATGGGACAGAA
β-Act R1^a^	TCCATGTCATCCCAGTTGCT

*^a^The primers were designed with the software Primers3Plus online.*

The *L. virgata* EPSPS cDNA sequences information can be found in GenBank with accession numbers KX425854 and KX425855.

### EPSPS Gene Expression

Young leaf samples (≈100 mg) from six untreated plants of both S and R15 populations were taken before treatment. Plants were then treated with 360 g ae ha^-1^ of glyphosate in the conditions used in the dose-response assays, and new samples were collected at 24 HAT. In both cases, the samples were stored at -80°C for RNA extraction and cDNA synthesis in the same conditions described in the previous section. The qLvFor1 and qLv-Rev1 primers (**Table 1**) to amplify a fragment of 114 bp were designed from the EPSPS gene sequences obtained in the previous section. The β-Actin and ADP-ribosylation factor genes were used as reference genes. Pairs of β-Actin primers (**Table 1**) were designed on the basis of the *Agrostis stolonifera* (JX644005.1), *Avena sativa* (KP257585.1), *Lolium multiflorum* (AJ585201.1), *Triticum monococcum* (AF326781.1) and *Zea mays* (U60510.1) sequences from GenBank. β-Actin and qLv primers were designed using Primers3Plus. The ADP-RF(a) primers designed by Giménez et al. (2011) were used to amplify the ADP-ribosylation factor gene (**Table 1**). For each quantitative RT-PCR reaction 40 ng of cDNA, 10 μL PerfeCTa SYBR Green FastMix ROX (Quanta Bioscience), and 0.2 μM of both forward and reverse primers in a 15 μL final reaction volume were used. The PCR conditions were: initial cycle at 94°C for 5 min; 40 cycles of 94°C 30 s and 62°C 1 min. The PCR reactions were carried out using an ABI Prism 7500 sequence detection system (Applied Biosystems, Foster City, CA, USA). Three to four technical replications per plant were carried out in a factorial design of two glyphosate treatments and two populations.

The PCR efficiency for each pair of primer and sample was determined by the program qPCR data analysis LinRegPCR (version 11) according to Ruijter et al. (2009) using raw fluorescence as input data. Expression level of both the reference and target genes for each sample was determined with the follow equation:

(1) $$\begin{array}{c} N_{0}=0.2/E^{Cq} \end{array}$$

Where *N_0_* is expression of the gene, *E* is the PCR efficiency for each primer, *Cq* is the number of cycles needed to reach 0.2 arbitrary units of fluorescence. The mean PCR efficiency for each gene, population and treatment was determined according to Giménez et al. (2011). The stability of the expression of the reference genes (β-Actin and ADP-ribosylation factor) and Normalization Factor (NF) were determined using geNorm software for each sample according to Vandesompele et al. (2002).

### Statistical Analysis

The percentages data of dry weight reduction, survival and EPSPS enzyme activity were submitted to a non-linear regression analysis. The dose of glyphosate needed to reduce the weight of a population (ED_50_), mortality (LD_50_), and to inhibit EPSPS activity (I_50_) by 50% were calculated. A log-logistic model of four parameters was conducted using the *drc* statistical package (Ritz et al., 2015) in the program R version 3.2.5. The statistical model is:

(2) $$\begin{array}{c} Y=c+\{(d-c)/[1+{\left(x/g\right)}^{b}]\} \end{array}$$

Where *Y* is the percentage of dry weight, survival and/or EPSPS-inhibiting with respect to the control, *c* and *d* are coefficients corresponding to the upper and lower asymptotic limits, *b* is the Hill slope, *g* is the glyphosate dose (ED_50_, LD_50_, or I_50_) at the mean point of inflection between the upper and lower asymptote and *x* (independent variable) corresponds to the glyphosate dose. The data were plotted using SigmaPlot 11.0 (Systat Software, Inc., USA).

EPSPS normalized expression level was calculated for each qPCR reaction, the average and standard error from technical replicates were recorded for each plant and population.

Absorption, translocation and EPSPS expression results were subjected to ANOVA using Statistix version 9.0 from Analytical Software (Tallahassee, FL, USA). When necessary, the means were compared using Tukey’s test’s at the 95% probability level.

## Results

### Glyphosate Dose–Response

**FIGURE 1:**
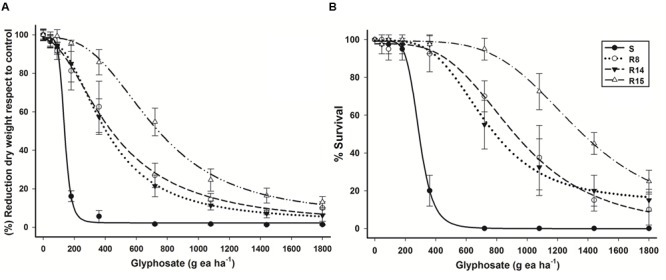
**Log–logistic curves of glyphosate-susceptible and -resistant *L. virgata* populations evaluated at 21 DAT. (A)** Dose–response curve with respect to percentage of dry mass reduction. **(B)** Dose–response curve with respect to percentage of survival. Vertical bars represent the standard error of the mean (*n* = 10).

Glyphosate resistance was confirmed in the three *L. virgata* populations (R8, R14, and R15) collected in Persian lime groves from Veracruz. These glyphosate resistant populations were less sensitive to glyphosate than the S population (**Figure 1**). R8 and R14 populations had similar response to glyphosate (**Figure 1A**). The resistant index (RI) of the glyphosate resistant populations ranged between 2.9 to 5.2 with respect to S population (**Table 2**).

**Table 2:** Parameters of the sigmoidal equation used to estimate ED_50,_ LD_50_ and I_50_ values of the glyphosate-susceptible and resistant *L. virgata* populations.

Population	c	d	b	*R*^2^aj	Mean dose^a^ (CI 95%)	RI^b^
**Parameters of ED_50_ value (g ae ha^-1^)^c^**						
S	2.28	98.34	6.62	0.99	137.7 (129.2, 146.1)	
R8	0.70	97.69	1.94	0.97	447.6 (403.5, 491.8)	3.3
R14	2.80	98.17	2.33	0.98	402.0 (371.2, 433.0)	2.9
R15	5.67	98.49	2.90	0.98	719.8 (668.2, 771.4)	5.2

**Parameters of LD_50_ value (g ae ha^-1^)^c^**						
S	-0.07	99.19	6.47	0.99	291.3 (263.7, 318.7)	
R8	0.10	97.80	3.52	0.95	932.3 (777.0, 1087.6)	3.2
R14	13.94	99.51	3.79	0.95	724.4 (624.9, 823.0)	2.5
R15	6.97	99.18	4.57	0.96	1330.3 (1084.2, 1576.4)	4.6

**Parameters of I_50_ value (μM)^d^**						
S	0.12	100.0	2.9	0.99	0.32 (0.04, 0.59)	
R8	0.10	100.0	11.0	0.99	0.67 (0.12, 1.22)	2.1
R14	0.11	100.0	13.0	0.99	0.50 (0.16, 0.84)	1.6
R15	1.64	100.2	1.2	0.99	53.09 (30.39, 75.78)	165.9

*c = lower limit, d = upper limit, b = Hill’s slope, R^2^aj = 1 - (sums of squares of the regression/corrected total sums of squares). ^a^Effective mean dose required: ED_*50*_ = to reduce the dry biomass by 50%; LD_*50*_ = to kill 50% plants of a population; and I_*50*_ = to reduce the EPSPS enzyme activity by 50%. ^b^RI = Resistance index (R/S) calculated using the corresponding ED_*50*_, LD_*50*_ or I_*50*_ values of the resistant populations respect to the susceptible one. ^c^CI values are the 95% confidence intervals (*n* = 10). ^d^CI values are the 95% confidence intervals (*n* = 3).*

Based on 50% mortality (LD_50_), the R8, R14 and R15 populations were, respectively, 2.5, 2.3, and 4.6 times more resistant than the S population (**Figure 1B**). A field dose of glyphosate (720 g ae ha^-1^), used in Persian lime groves of Veracruz, was enough to achieve full control in the S population. However, with this dose of glyphosate, only 50% mortality was observed in R8 and R14 populations, and to the R15 for population, a dose 1.8 times more glyphosate than the field dose was required to obtain the same level of control. The ED_50_ and LD_50_ parameters showed the different resistance levels to glyphosate acquired by resistant *L. virgata* populations (**Table 2**).

### Shikimic Acid Accumulation

**FIGURE 2:**
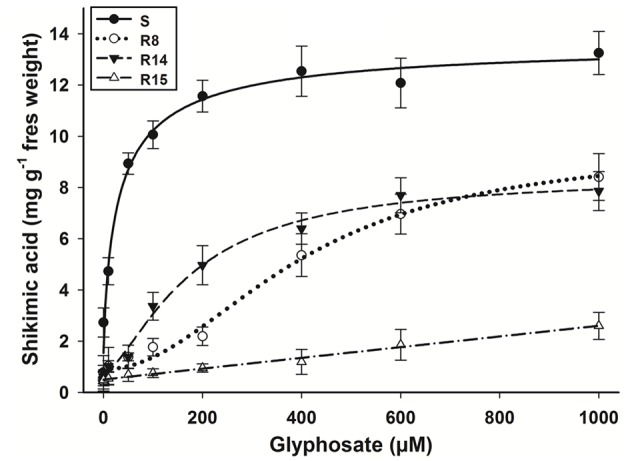
**Shikimic acid accumulation of glyphosate-susceptible and -resistant *L. virgata* populations at different glyphosate concentrations.** Vertical bars represent the standard error of the mean (*n* = 3).

The amounts of shikimic acid accumulated after glyphosate treatment is highly variable between species and populations. In this work, the shikimic accumulation was different in each *L. virgata* population. The S population presented the highest level of shikimic acid accumulation (**Figure 2**). This accumulation was exhibited at lower glyphosate concentrations (between 0.1 to 100 μM) reaching an average of 12.35 mg shikimic g^-1^ fresh weight from 200 μM of glyphosate. The resistant populations only presented an appreciable accumulation from 100 μM of glyphosate. The averages of shikimic acid accumulated were 8.4, 7.9, and 2.6 mg shikimic g^-1^ fresh weight for the R8, R14, and R15 populations, respectively, at the glyphosate concentration of 1000 μM.

The response patterns to glyphosate in shikimate accumulation assays were similar to that observed in the dose-response study. The lowest shikimate accumulation observed in the R15 population compared to the R8 and R14 populations was consistent with lower growth reduction and mortality observed in the plants (**Figures 1** and **2**).

### Absorption and Translocation

**FIGURE 3:**
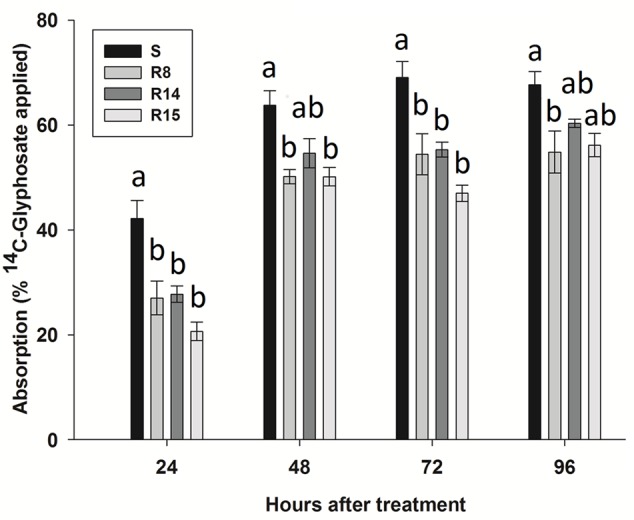
**^14^C-glyphosate absorption in glyphosate-susceptible and -resistant plants of the *L. virgata* populations.** Different letter at each evaluation time is statistically different at 95% probability determined by the Tukey’s test. Vertical bars represent the standard error of the mean (n = 5).

The four *L. virgata* populations presented a high absorption index of ^14^C-glyphosate, absorbing amounts of over 50% from recovered herbicide at 96 HAT (**Figure 3**). At 24 HAT, the S population presented the highest ^14^C-glyphosate absorption rate. Between 48 to 96 HAT, glyphosate absorption was similar among populations, ranging from 47.7 to 67.7%. However, R15 population showed a clear reduced ^14^C-glyphosate absorption in comparison to the S population (**Figure 3**).

**FIGURE 4:**
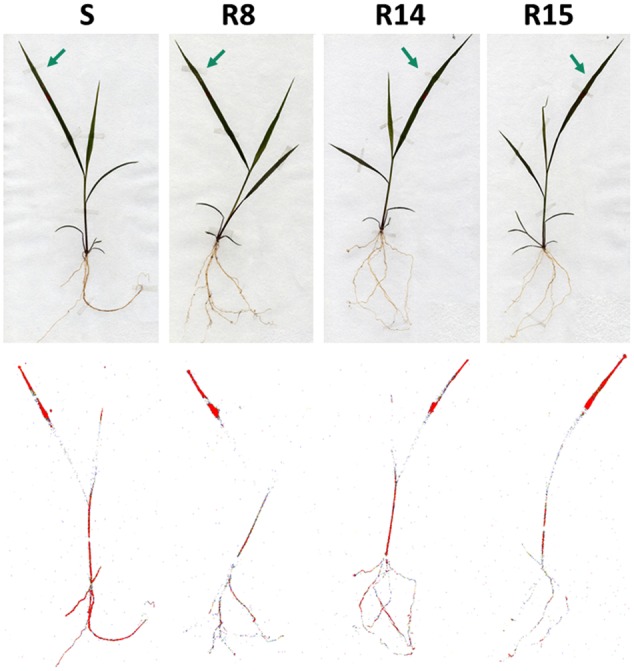
**Digital images (top row) and autoradiograph images (bottom row) of ^14^C-glyphosate translocation in glyphosate-susceptible and -resistant plants of *L. virgata* populations.** The autoradiograph images were obtained from treated plants at 96 HAT. The highest concentration of ^14^C-glyphosate is highlighted in red. Arrows indicate the treated leaf.

*L. virgata* populations showed similar ^14^C-glyphosate translocation patterns. In the aerial part of the plant (treated leaves and rest of plant). However, the greatest differences of ^14^C-glyphosate translocation were found in the roots. At 96 HAT, R15 population had the lowest rate of translocation to the root (21.9% of the absorbed herbicide), translocating ±10% less herbicide than the S population. The R8 and R14 populations translocated an average of 24.5 and 26.5%, respectively, of herbicide translocated to the roots at 96 HAT (**Table 3**). At this time, the S population had already reached a balanced distribution of herbicide between treated leaf, rest of plant and roots. The Phosphor Imager images confirmed the previous results of ^14^C-glyphosate translocation. At 96 HAT, the plants of resistant populations, mainly the R15 population ones, translocated smaller amounts of ^14^C-glyphosate from treated leaf to the root than the S population plants (**Figure 4**).

**Table 3:** Translocation percentage of ^14^C-glyphosate in plants of glyphosate-susceptible and -resistant *L. virgata* populations.

Population	HAT	Translocation (% from absorbed)^a^		
		Treated leaf	Rest of plant	Root
S	24	75.1 ± 2.0 ab	16.0 ± 0.8 h	8.9 ± 2.6 j
	48	55.7 ± 2.6 c	24.2 ± 2.0 ef	20.2 ± 1.3 defg
	72	44.7 ± 1.0 de	30.3 ± 1.4 bcd	25.0 ± 1.1 bc
	96	33.9 ± 1.9 g	33.9 ± 1.0 ab	32.2 ± 1.2 a

R8	24	74.5 ± 2.1 ab	16.8 ± 1.5 gh	8.7 ± 1.2 j
	48	55.1 ± 1.9 c	27.9 ± 1.8 cde	17.0 ± 2.0 fgh
	72	45.3 ± 2.4 de	33.8 ± 2.6 ab	20.9 ± 1.5 cdef
	96	38.2 ± 1.1 fg	37.3 ± 3.8 a	24.5 ± 2.6 bcd

R14	24	70.3 ± 0.9 b	18.8 ± 1.1 fg	10.9 ± 0.4 ij
	48	56.4 ± 1.9 c	27.5 ± 1.0 cde	16.0 ± 2.0 gh
	72	43.5 ± 2.3 def	36.2 ± 1.2 a	20.3 ± 2.1 cdefg
	96	39.8 ± 2.8 ef	33.7 ± 3.5 ab	26.5 ± 1.0 b

R15	24	78.0 ± 2.2 a	12.3 ± 1.1 gh	9.6 ± 1.3 j
	48	58.7 ± 1.7 c	26.4 ± 1.7 de	14.9 ± 0.8 hi
	72	48.7 ± 2.2 d	32.3 ± 1.0 abc	19.0 ± 1.6 efgh
	96	43.2 ± 1.5 def	34.9 ± 1.5 ab	21.9 ± 2.2 cdef

*^a^Means with different letter within a column are statistically different at 95% probability determined by the Tukey’s test. ±SEM (*n* = 5).*

### Enzyme Activity

**FIGURE 5:**
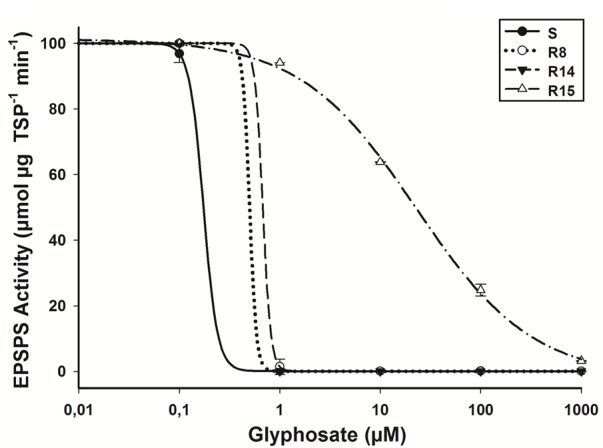
**EPSPS enzyme activity expressed as percentage of the untreated control in leaf extracts of plants from glyphosate-susceptible and resistant *L. virgata* populations.** Vertical bars represent the standard error of the mean (*n* = 3).

In the absence of glyphosate, significant differences (*P* = 0.035; *DF* = 3; *n* = 12) in the basal EPSPS activity between plants of *L. virgata* populations were found. R15 population presented an average of 0.41 μmol μg TSP^-1^ min^-1^, while the S, R8, and R14 populations an average of 0.29 μmol μg TPS^-1^ min^-1^. The EPSPS enzyme activity was inhibited by glyphosate in plants from susceptible and resistant populations while the concentrations increased. To inhibit EPSPS activity by 50% (I_50_) for the S population was required 0.32 μM of glyphosate (**Table 2**). Plants from the R15 population showed an RI of 165.9 higher with respect to the S population plants (**Figure 5**). According to the confidence intervals (CI95%), R8, R14, and S population plants showed no significant differences in their EPSPS enzyme activity.

### EPSPS Sequencing and Gene Expression

**FIGURE 6:**
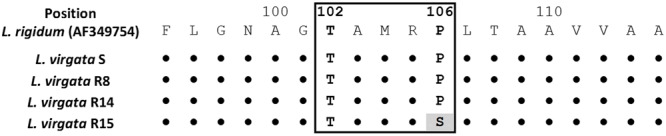
**Partial alignment of protein sequences of EPSPS gene in glyphosate-susceptible and -resistant *L. virgata* populations.** The highlighted color indicates a change at 106 position from CCA (Proline) to TCA (Serine) in the consensus nucleotide sequence. Box includes from the 102 to 106 positions (amino acid number based on the start codon of *L. rigidum* [GenBank: AF349754] EPSPS sequence), corresponding to point mutations associated for conferring glyphosate resistance. The *L. virgata* EPSPS cDNA sequences information can be found in GenBank with accession numbers KX425854 and KX425855.

A fragment of 559 bp in length were amplified included the Thr-102 and Pro-106 positions in the protein sequence. The predicted amino acid sequence of S population presented as the same consensus of *Lolium rigidum* (GenBank: AF349754), and others grass glyphosate susceptible species. In the Thr-102 position no mutation was found. Only the R15 population showed a codon change from CCA to TCA resulting in an amino acid substitution from Proline to Serine at 106 position (**Figure 6**).

No significant differences (*P* = 0.6924; *DF* = 3; *n* = 12) were found in the EPSPS expression in untreated (0 HAT) plants used as a control between populations. The S and R15 populations showed an increased in the EPSPS expression level after glyphosate application at 24 HAT, but it was similar in both populations, with an average of 3.84 times more expression of the EPSPS (**Table 4**).

**Table 4:** EPSPS expression level in treated and untreated plants of the glyphosate-susceptible and resistant *L. virgata* populations.

Population	Expression level^a^ (EPSPS:NF^b^)	EI^c^	EPSPS expression level (0 HAT:24 HAT)^d^	EI^c^
S	4.67 ± 0.50		4.02 ± 0.61	
R15	4.50 ± 0.53	0.96	3.67 ± 0.44	0.91

Mean	4.58 ± 0.49		3.84 ± 0.54	

*^a^Values (×10^*3*^) obtained from untreated (0 HAT) plants. ^b^NF = Normalization Factor. ^c^Expression index EI = Expression level R/Expression level S. ^c^The coefficients of expression were estimated as: Expression level at 24 HAT/Expression level at 0 HAT of each population. ±SEM (*n* = 6).*

## Discussion

The index resistance (IR) of resistant *L. virgata* populations ranged from 2.9 to 5.2 times more than the S population. Previously, *L. virgata* was effectively controlled with glyphosate in citrus orchards of Veracruz with 720 g ae ha^-1^ (field dose), until the identification of the first resistant populations (Pérez-López et al., 2014). The LD_50_ values estimated in the resistant populations studied in this work were higher than those at the field glyphosate dose. Other glyphosate-resistant grass weeds such as: *Bromus diandrus, Echinochloa colona, Eleusine indica, Lolium perenne* spp. *multiflorum, L. rigidum, Poa annua*, among others (González-Torralva et al., 2012; Alarcón-Reverte et al., 2015; Chen et al., 2015; Cross et al., 2015; Fernandez et al., 2015; Salas et al., 2015; Yu et al., 2015; Malone et al., 2016), exhibited RI values that ranged between 3 to 19, and between 4 to <182 based on in their ED_50_ or LD_50_, respectively. Differences in the level of glyphosate resistance between these species were due to various resistance mechanisms.

Both shikimic acid accumulation and dose-response results indicated that the R8 and R14 populations of *L. virgata* possessed similar levels of glyphosate resistance, and the R15 population was the most resistant one. R8 and R14 populations had similar glyphosate application history (3–4 times per year for 8–10 years), despite coming from different geographical places. R15 population was subjected to 3–4 application per year for over 15 years (Pérez-López et al., 2014), in a citrus grove geographically close to the grove where R14 population was collected (Alcántara-de la Cruz et al., 2016c). It is evident that the selection pressure exerted by glyphosate in the R15 population caused low sensitivity to the herbicide.

Glyphosate absorption is a biphasic process. First the glyphosate should penetrate rapidly through the cuticle, and then is slowly absorbed through the phloem (Gomes et al., 2014). Reduced absorption is a mechanism not usually involved in the glyphosate resistance and has been described in a few species such as *D. insularis, L. multiflorum*, and *S. halepense* (Michitte et al., 2007; de Carvalho et al., 2012; Vila-Aiub et al., 2012). Yanniccari et al. (2012) suggested that there is an unidentified barrier that prevents glyphosate to load into the phloem. In the resistant *L. virgata* populations, the reduced glyphosate absorption played an important role, mainly in the first 24 HAT, due to a clear reduction compared to the S population. The absorption process time, depends on the treated species, age of the plant, herbicide concentration and environmental conditions (Gomes et al., 2014). Considering that the populations were studied in similar controlled conditions, it is clear that resistant *L. virgata* populations have limited the foliar absorption of glyphosate, mainly in the first hours after treatment, where the S population absorbs up to twice the herbicide that the most resistant population (R15). Thus, the tropical rainy conditions of the citrus region of Veracruz end up favoring resistant *L. virgata* populations, reducing the amount of glyphosate that could be absorbed by the treated plants and reach the target-site. Similar patterns of reduced absorption were observed in *B. pilosa*, where the S population absorbs at least twice more herbicide that the resistant populations in the first 24 HAT (Alcántara-de la Cruz et al., 2016b).

Different glyphosate concentrations in the tissue are related to differences in glyphosate efficacy (Alarcón-Reverte et al., 2015). Glyphosate will reach active metabolic sites, such as root and shoot meristems to act (Gomes et al., 2014). Greater movement of glyphosate to the meristematic tissues is crucial for plant mortality (Adu-Yeboah et al., 2014). However, reduced glyphosate translocation in resistant plants is mostly retained within the treated leaves (Yanniccari et al., 2012). Plants of the resistant *L. virgata* populations showed low glyphosate translocation to the meristematic tissues of the roots. The lowest one being exhibited by the R15 population. Reduced glyphosate translocation is due to a trait (unknown barrier) that restricts glyphosate movement within resistant plants (Powles, 2010), or an altered subcellular distribution of glyphosate, keeping it away from the target-site (Kleinman and Rubin, 2016). The barrier may exist either in the phloem system or in the mesophyll cells, where glyphosate must enter to be translocated (Yanniccari et al., 2012). Glyphosate resistance by this mechanism confer by a single nuclear gene with complete- or semi-dominance (Shaner, 2009). R15 population was selected by glyphosate during a longer time period, this explains why it has the lowest translocation rate. However, the genetic determinants of NTSR continues to be poorly know (Déyle, 2013). Reduced translocation has been reported as being a mechanism responsible for endow glyphosate resistance in different grass weed species such as *D. insularis, L. multiflorum, L. perenne, L. rigidum, Sorghum halepense* (Bostamam et al., 2012; de Carvalho et al., 2012; González-Torralva et al., 2012; Vila-Aiub et al., 2012; Adu-Yeboah et al., 2014; Fernandez et al., 2015; Ghanizadeh et al., 2015), among others. In some cases, it was reported as a major resistance mechanism (Adu-Yeboah et al., 2014). Glyphosate resistant biotypes of *C. bonariensis* showed an altered subcellular distribution of glyphosate (Kleinman and Rubin, 2016). We suggest that *L. virgata* developed reduced absorption and translocation as resistance mechanisms against glyphosate first, independently of their geographic or genetic relationship.

The different shikimic acid accumulation, reduced absorption and translocation patterns presented by the R15 population, suggest that their glyphosate resistance mechanism may differ from that of the R8 and R14 populations. In addition, reduced glyphosate absorption by plants and higher EPSPS activity, have been associated with a decreased sensitivity to glyphosate (Salas et al., 2012).

On the other hand, EPSPS enzyme activity tests, in addition to any other appropriate parameter to determine glyphosate resistance (Dayan et al., 2015), also allows us to suspect the possible mechanisms that may be involved in the target-site. A greater basal activity is often associated with a larger number of copies or overexpression of the EPSPS gene (Salas et al., 2012). Also, a greater content EPSPS protein, allows that some molecules act as sponge to absorb glyphosate, while others molecules continue their essential function in the shikimic pathway (Powles, 2010). The R15 population presented the highest EPSPS content among the *L. virgata* populations. Gene amplification is a well-characterized phenomenon in plant evolution (Powles, 2010). However, the R15 population of *L. virgata* evolved under anthropogenic selection pressures, showing an enhanced EPSPS basal activity that allows to cope with the presence of glyphosate. The EPSPS of glyphosate-resistant plants could be equally sensitive to glyphosate (Powles, 2010). This happens when the EPSPS gene does have not mutations in the Thr-102 and Pro-106 positions yet. The R8 and R14 populations of *L. virgata*, showed no significant differences in their inhibition of the EPSPS enzyme by glyphosate with respect to the S population. Moreover, these populations showed no mutation in the EPSPS gene. This suggests that their resistance mechanism did not involve the target-site. Similar results were reported in resistant populations of *E. indica* (population R3) and *E. colona* (population RLB2) (Alarcón-Reverte et al., 2015; Chen et al., 2015), in which no important differences in their inhibition of the EPSPS activity with respect to their sensitive populations were reported.

Therefore, the scant inhibition of EPSPS enzyme activity by glyphosate in the R15 population, also presented a mutation found in the Pro-106 position of EPSPS gene, that provides of a higher resistance to glyphosate. Amino acid substitution at Pro-106 position in the EPSPS gene from Proline to Serine, Alanine, Threonine and/or Leucine have been widely reported to partially confer resistance to glyphosate, and often are accompanied by another mechanism (Sammons and Gaines, 2014). Some species which presented a mutation in combination with other resistance mechanisms (either reduced absorption, reduced translocation, multiple EPSPS gene copy number, overexpression of the EPSPS gene, and even metabolism) are: *A. palmeri. A. tuberculatus, B. diandrus, B. pilosa, D. insularis, E. colona, E. indica, L. perenne* sp. *multiflorum, L. rigidum, K. scoparia* (Bostamam et al., 2012; de Carvalho et al., 2012; González-Torralva et al., 2012; Alarcón-Reverte et al., 2015; Chatham et al., 2015; Chen et al., 2015; Fernandez et al., 2015; Salas et al., 2015; Wiersma et al., 2015; Alcántara-de la Cruz et al., 2016b; Malone et al., 2016).

Due to only the R15 population showed a greater basal activity, the EPSPS expression was studied in this resistant population in comparison to the S population. However, our qPCR analysis from cDNA (**Table 4**), indicated that the greater EPSPS basal activity of the R15 population, cannot be ascribed to an overexpression of the EPSPS gene. Alarcón-Reverte et al. (2015), suggested that it could have been an enhanced basal EPSPS activity as an additional TSR against glyphosate, possibly due to post-transcriptional regulation mechanisms increased mRNA stability and/or reduced enzyme degradation, as a consequence of the selection pressure exerted by glyphosate. A higher number of EPSPS gene copies correlate positively with higher EPSPS transcription (Gaines et al., 2013; Vila-Aiub et al., 2014). However, the manifestation of more EPSPS gene copies does not always result in differences in the amount of protein content (Salas et al., 2015). It is possible that the EPSPS copy number content of the R15 population could be greater than of the S population. However, we did not study this parameter among S and R15 populations, because no difference in the expression of the EPSPS gene was observed. Resistant populations of *A. palmeri, A. tuberculatus. B. diandrus, E. colona, E. indica, K. skoparia, L. perenne* spp. *multiflorum* (Salas et al., 2012; Gaines et al., 2013; Ribeiro et al., 2014; Alarcón-Reverte et al., 2015; Chatham et al., 2015; Chen et al., 2015; Wiersma et al., 2015; Malone et al., 2016), are examples of species which presented differences in the EPSPS copy numbers and/or overexpression of the EPSPS gene as glyphosate resistance mechanisms, in some cases as major ones. This results indicates that the R15 population presented NTSR and TRS mechanisms against glyphosate: reduced absorption, reduced translocation, enhanced EPSPS basal activity, and target-site mutation.

Glyphosate metabolism could contribute to natural tolerance. However, there is no evidence that metabolism plays a significant role in resistance to glyphosate. It could be developed due to an extreme selection pressure, but only when treated plants did not evolve glyphosate resistance via other mechanisms (Duke, 2011). We analyzed *L. virgata* aerial and roots tissue of plants randomly of the four populations treated with 360 ae ha^-1^ collected at 4 and 8 DAT, following the methodology used by de Carvalho et al. (2012). Resistant *L. virgata* plants analyzed did not show glyphosate metabolism as resistance mechanism.

According with the results of this work, the glyphosate resistance of *L. virgata* is a consequence of the selection pressure exerted by herbicide, and it did not present innate glyphosate tolerance. In addition, *L. virgata* was effectively controlled previously with glyphosate (Pérez-López et al., 2014; Alcántara-de la Cruz et al., 2016c). According to bibliography, innate tolerance to glyphosate not involve target site tolerance mechanisms within the same species, and the tolerant populations generally are compared with sensitive populations of other species (Yuan et al., 2002; Alcántara-de la Cruz et al., 2016a; Fernández-Moreno et al., 2016; Mao et al., 2016). To demonstrate the innate glyphosate tolerance in species such as: *Avena sterilis, Cologania broussonetii, Dicliptera chinensis, Liriope platyphylla, Liriope spicata, Ophiopogon japonicas*, among others, it has been necessary compare them with glyphosate sensitive species.

## Conclusion

The reduced absorption and translocation are the main mechanisms of resistance of *L. virgata* to glyphosate. In addition, an enhanced EPSPS basal activity and a mutation in the glyphosate target-site endow a high level of resistance in this species. These results demonstrate that *L. virgata* evolves similar glyphosate resistance mechanisms like many other weeds, independently of agronomic system.

To prevent the resistance spread of *L. virgata* across citrus groves, the growers must properly clean agricultural implements. Given the current global scale of glyphosate use, current field trials are being carried out by this research group, to find chemical and non-chemical alternatives, which are economically and environmentally viable, to integrate *L. virgata* management where this weed is common.

## Author Contributions

RA-C, HC-H, FB, JD-V, and RDP: Idea and designed the experiments; RA-C, AR-D, and MG: Performed the research; RA-C, AR-D, and MG: Interpretation and analysis of results (of raw data); RA-C, AR-D, MG, HC-H, FB, JD-V, and RDP: Wrote and approved the manuscript.

## Conflict of Interest Statement

The authors declare that the research was conducted in the absence of any commercial or financial relationships that could be construed as a potential conflict of interest.

## Footnotes

1 http://www.bioinformatics.nl/cgi-bin/primer3plus/primer3plus.cgi
